# A sterilization method for human decellularized vaginal matrices

**DOI:** 10.1038/s41598-024-82409-4

**Published:** 2024-12-30

**Authors:** Jayson Sueters, Leonie de Boer, Freek Groenman, Judith A. F. Huirne, Theo H. Smit, Sebastian A. J. Zaat

**Affiliations:** 1 Department of Gynaecology, Amsterdam UMC – Location VUmc, De Boelelaan 1117, 1081 HV Amsterdam, The Netherlands; 2 Department of Medical Microbiology and Infection Prevention, Amsterdam UMC – Location AMC, Meibergdreef 9, 1105 AZ Amsterdam, The Netherlands; 3 Department of Obstetrics and Gynecology, Amsterdam Reproduction and Development, Amsterdam UMC – Location VUmc, De Boelelaan 1117, 1081 HV Amsterdam, The Netherlands; 4 Department of Medical Biology, Amsterdam UMC – Location AMC, Meibergdreef 9, 1105 AZ Amsterdam, The Netherlands; 5 Amsterdam Reproduction and Development Research Institute, Amsterdam UMC, Meibergdreef 9, 1105 AZ Amsterdam, The Netherlands; 6 Centre of Expertise on Gender Dysphoria, Amsterdam UMC – Location VUmc, De Boelelaan 1117, 1081 HV Amsterdam, The Netherlands

**Keywords:** Decellularization, Sterilization method, Vagina, Human acellular matrix, Transplant immunology, Implants, Biomaterials

## Abstract

Vaginal reconstruction is necessary for various congenital and acquired conditions, including vaginal aplasia, trauma, tumors, and gender incongruency. Current surgical and non-surgical treatments often result in significant complications. Decellularized vaginal matrices (DVMs) from human tissue offer a promising alternative, but require effective sterilization to ensure safety and functionality. This study aimed to develop a sterilization method for decellularized human vaginal wall scaffolds. Based on our previously implemented decellularization technique with minor modifications, we designed and examined three sterilization methods consisting of (i) chemical decellularization, (ii) decellularization with additional peracetic acid/hydrogen peroxide (PAA/H_2_O_2_); (iii) decellularization with antibiotic and antimycotic (AAE) based treatment. Sterilization efficacy was evaluated through controlled contamination with common vaginal microbes and sterility testing subsequent to each sterilization method. The extracellular matrix (ECM) structure was assessed via histological staining. Decellularization alone reduced some added bacterial contaminants but did not achieve complete sterilization. PAA/H_2_O_2_-sterilization resulted in severe ECM damage, rendering it unsuitable. The AAE-treatment demonstrated effective sterilization without compromising the ECM structure. Combined decellularization and AAE-based treatment forms a viable sterilization method for human vaginal wall tissue, maintaining ECM integrity and achieving effective micro-organism elimination. This method holds potential for clinical application in vaginal transplantation.

**Supplementary Information:**

The online version contains supplementary material available at 10.1038/s41598-024-82409-4.

## Introduction

The vagina wall is a fibromuscular tube, that plays an essential role in sexual intercourse, discharge of menstrual blood and in childbirth^1^. Vagina reconstruction is needed for many congenital or acquired diseases, including vaginal aplasia, trauma, tumor growth (with radiation damage) and feminine gender incongruency^2^. Several surgical and nonsurgical treatments are available, but these are all associated with serious drawbacks^3–5^. Long-term nonsurgical dilation might not be optional and causes pain, mental and emotional distress and high risk of stenosis^3,4,6,7^, whilst surgical treatments carry risk of stenosis, necrosis, vaginal prolapse, and donor site morbidity^5,8–12^. Furthermore, surgery with use of a non-vaginal graft results in complications and complaints related to dyspareunia^13–15^, insensibility^16,17^, and excessive or lack of vaginal discharge^18,19^. Therefore, alternative treatment options based on transplantation or tissue engineering techniques are explored.

Approximately 90,000 whole organ transplantations are performed worldwide each year^20^. Amongst transplantation, a 1% occurrence of transmitted infections is reported, but these are generally only recognized after clusters of infections are observed in recipients with a common donor. Furthermore, in case of transmitted infections the risks are severe and include sepsis, graft rejection, and postoperative morbidity and mortality^20^. Thus, success of transplantation relies on effective sterilization. In answer to a shortage of organ donors, recent developments in the field of tissue engineering and regenerative medicine have resulted in promising new biomaterials for tissue replacement of various organs^21^, including the vagina. Specifically for reconstruction of the vagina, decellularized matrices from skin^22–24^, small intestinal submucosa^25–30^ (SIS) and urinary bladder matrix^31,32^ (UBM) have been applied. However, xenogeneic-derived decellularized matrix may cause severe immunologic reactions^33^ and autologously-derived materials may cause donor site morbidity^34^. Alternatively, decellularized vaginal matrix (DVM) could be used for vagina reconstruction^1,35–37^, which is an extracellular matrix (ECM)-derived bioscaffold. In a previously conducted study, we have developed a decellularized matrix from healthy human vagina donor tissue conform established criteria for acellular biomaterials^2^. Compared with previously mentioned biomaterials, DVM can promote formation of neo-vagina rapidly^1^.

Our method and several other decellularization procedures result in acellular vaginal wall tissue with retained biomechanical integrity, important ECM proteins, and tissue aspects essential for cell adhesion, survival and proliferation^1,35–37^. Although current immunosuppression therapy has improved the chance of graft survival by preventing tissue rejection, clinical application of tissue-engineered transplants in immunocompromised hosts is challenging due to a higher susceptibility to infections. Therefore, a terminal sterilization method prior to implantation is required^38^. The vaginal microbiota contains vaginal *Lactobacillus* species (*Lactobacillus crispatus*, *Lactobacillus iners*, *Lactobacillus gasseri* or *Lactobacillus jensenii*)^39–43^. In the human vaginal microbiota typically also a predominance of *Actinobacteria*, *Prevotella*, *Veillonellaceae*, *Streptococcus*, *Probacteria*, *Bacteroides*, *Bifidobacteriaceae*, and *Burkholderiales* is observed^42^. However, many aspects can temporally change the vaginal microbiota, causing differences among individuals due to variation in sexual activity, douching, chronic stress, race, medication, diet, smoking or pregnancy^40,44^. Also an abnormal vaginal microflora may occur due to the presence of for example (sexually transmitted) infections i.e., *Streptococcus pneumoniae*, *Haemophilus influenzae*, *Listeria monocytogenes*, or due to an overgrowth of naturally occurring species such as *Escherichia coli* or *Staphylococcus aureus*^42,43^. Among non-bacterial species, *Candida albicans* is a well-known fungus to cause vaginal infections^44^. To minimize the risk of disease transmission to clinically acceptable levels, sterilization of biomaterials is required^45^. This extends their potential use beyond the sole treatment of life-threatening conditions, to patients who could benefit from their use for reconstruction of other organs^45^, such as vagina or skin. Sterilization of vaginal matrix would thus minimize risks and increase the clinical acceptability of human donor vagina.

A sterilization method should be validated according to a predefined applicable standard for that specific medical device or biomaterial. To this aim, an ideal sterilization method should be able to eliminate all bioburden present in a biomaterial and prevent rigorous changes to its biological and mechanical properties that could compromise the integrity and function of an implant^46^.

A multitude of validated processing methods have been published to render a biomaterial sterile. Gamma irradiation is known to cause major structural damage^47,48^. Ultraviolet (UV) irradiation has a weak penetration capacity and is ideal for surface disinfection, but unsuitable for use on liquid medicines and for internal disinfection of solids^46^. Hydrogen peroxide (H_2_O_2_) is a disinfectant that can achieve a sterilization effect under certain conditions^49^, and sterilization by hydrogen peroxide low-temperature plasma (HPLP) is a common method in hospitals^46^. However, H_2_O_2_ generates many active oxygen and high-energy free radicals, that cause cytomembrane disruption and denaturation of proteins and nucleic acids^50^. The strong oxidation may further affect the physical and chemical properties of biomaterials^51^. Ethylene oxide (ETO) is an effective sterilization agent, but requires the use of an ETO device and further produces toxic reaction products, such as ethylene chlorohydrin^52,53^. Both ETO and these reaction products can cause severe pathological changes in implants^45^. ETO has been classified as mutagen and carcinogen, which has caused strict regulations around its use for sterilization^45^. Sporadically, supercritical carbon dioxide (ScCO_2_) is used to disinfect or sterilize biomaterials. However, its exact effect on killing pathogens is unknown and ScCO_2_ may have effect on the physical and chemical properties of a biomaterial^54,55^, and could further cause partial dissolution of proteins by acidification^46^.

For these reasons we looked at an alternative sterilization procedure for human DVM. Peracetic acid (PAA) is a common disinfectant, but can also act as sterilizing agent with a demonstrated effect against bacteria, viruses, and spores^56–58^. Use of PAA has been approved by the Food and Drug Administration (FDA)^59^, and has been applied at concentrations of 0.05–0.1% for sterilization of a variety of organs (i.e., cardiac and arterial valves^60^, lung^61^, liver^62,63^, bladder^64–66^, spleen^67,68^, kidney^69,70^, small intestine^71–73^, tendon^74,75^ and nerve^76^) and biomaterial tissues (i.e., bone, heart valves, pericardium, liver, small intestine, lung, kidney, spleen, bladder, tendon and porcine vagina)^35,46^. PAA-sterilization does carry concerns about residual reactions after biomaterial implantation, and its potential hazard to PAA handling staff and the environment need to be considered^45,46^. Furthermore, the oxidation and acidity of PAA can potentially affect the physical and chemical properties of a biomaterial^64,73^.

Alcohol is a disinfectant known to cause denaturation of proteins^77^. However, short soaking (5 min to 4 h) of biomaterials in alcohol combined with other disinfectant and sterilization agents have previously resulted in successful sterilization of biomaterial scaffolds for reconstruction (i.e., pericardium^78^, heart valve^79^, blood vessel^80^, liver^81,82^, pancreas^83^, trachea^84^, bladder^83^, kidney^69^, bone^85^, tendon^86,87^ and nerve^88^). Long exposure to alcohol at high concentrations is further known to potentially denature proteins^89^. Antibiotics are not typically used for disinfection or sterilization, but can kill micro-organisms through various mechanisms^90^. The most common antibiotics applied for cell cultures, tissue engineering and regenerative medicine are penicillin and streptomycin. Penicillin inhibits synthesis of bacterial cell walls, whereas streptomycin prevents protein translation of bacteria through its binding to a ribosome subunit^46^. Amphotericin-B is an antimycotic agent often added to cell cultures (in combination with antibiotics), and inhibits the growth of fungi and some protozoa by affecting the permeability of cell membranes^46^. Antibiotics and antimycotics do not affect tissue properties, but streptomycin is one of the antibiotics that could cause toxic reactions in the human body at high residual concentrations^46^.

The aim of this study was to overcome limitations associated with previous sterilization methods, by developing a sterilization method for human DVM. Three methods were designed and tested: (i) sterilization by chemical decellularization^2^, (ii) sterilization by chemical decellularization and additional 0.01%, 0.05% or 0.1% PAA/H_2_O_2_ treatment and (iii) decellularization and additional mixed antibiotic, antimycotic and alcohol treatment. A 0.01–0.1% PAA range was examined to minimize structural damage. The developed protocols were histologically assessed in terms of structural extracellular matrix preservation. Furthermore, controlled microbial contamination of vaginal wall tissue was performed prior to decellularization combined with sterilization, and the induced level of sterility was evaluated.

## Materials and methods

### Surgical procedure

Vaginal tissue was retrieved from six healthy transmasculine donors during colpectomy at Amsterdam UMC (the Netherlands) between December 2023 and June 2024^2^. No tissue was procured from prisoners. As previously described, robotic-assisted laparoscopic colpectomy (daVinci XI system, Intuitive) was performed as part of the donor’s medical transition according to an established method^91^. Vaginal epithelium was dissected with monopolar scissors, and bleeding was minimized with fenestrated bipolar forceps^92^. Tissue from vaginal epithelium, lamina propria and smooth muscle was removed. Vaginal adventitia was preserved to prevent fistula to bladder, urethra or rectum, bleeding from perivaginal plexus and nerve injury to adjacent structures^92^. Per donor, three vaginal wall rings were obtained. After surgical retrieval, the vaginal tissue was directly stored in phosphate buffered saline (PBS—Fresenius Kabi), pH 7.4, on ice for a maximum of 4 h until decellularization. Written informed consent for experimental use of resected vaginal tissue was obtained from all donors. Tissue collection and experimental usage was approved by the institutional Medical Ethical Examination Committee of Amsterdam UMC location VU Medical Center (Amsterdam; IRB approval by METc VUmc registration number 2018/3190, October 2018) and performed in accordance with relevant guidelines and regulations.

### Experimental design

**Fig. 1:**
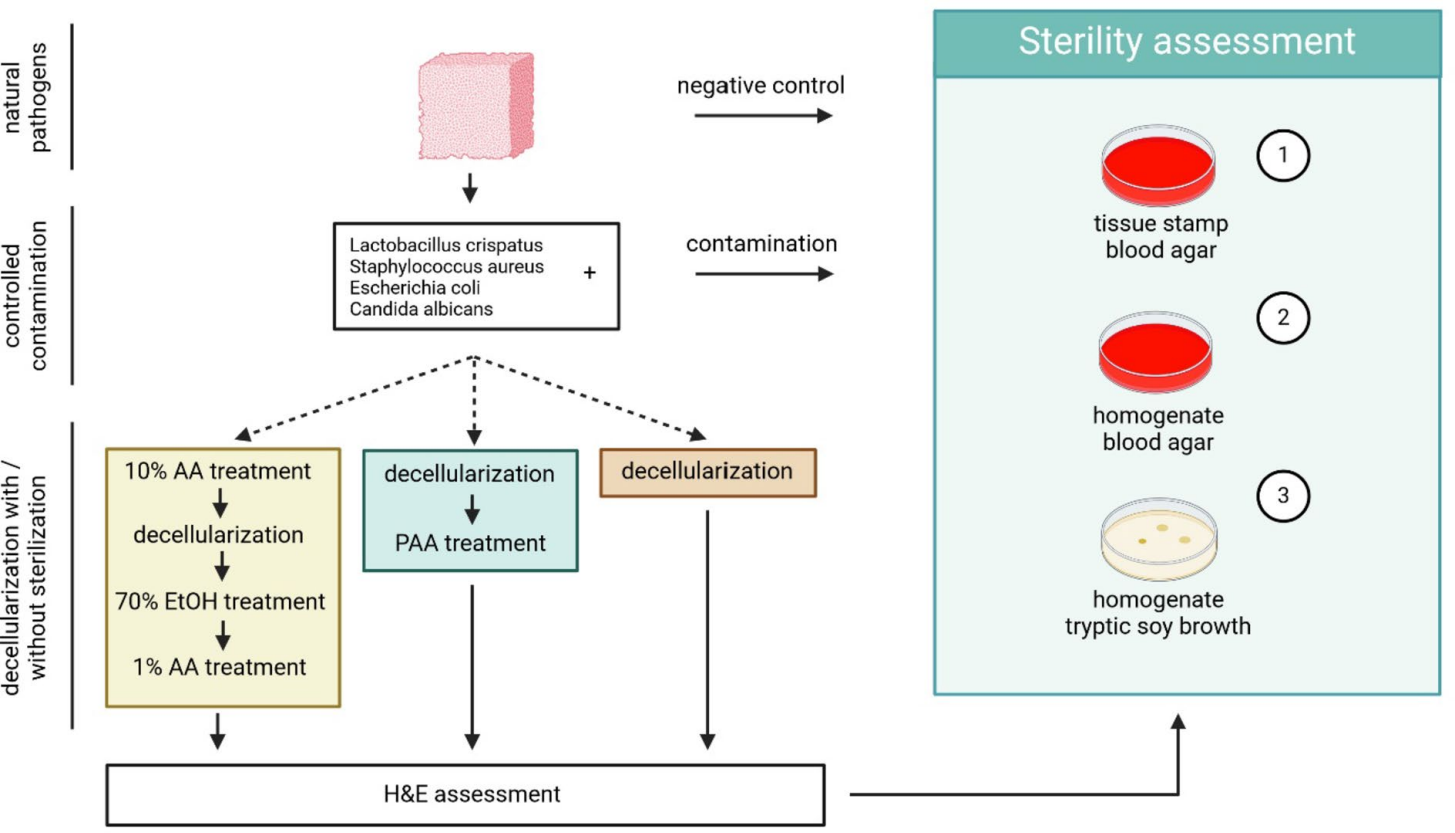
Flowchart assessment of sterilization efficacy. Naturally occurring micro-organisms in donated vaginal wall tissue were identified in negative controls. After controlled contamination with *L. crispatus*, *S. aureus*, *E. coli* and *C. albicans* strains at known concentrations, contamination was confirmed. The efficacy of decellularization with and without sterilization on these contaminants was assessed through blood agar plating of tissue stamps and tissue homogenate.

Decellularized vaginal scaffolds were prepared from retrieved human vaginal wall tissue utilizing a previously established protocol^2^. In order to validate sterilization efficacy, an extensive assessment protocol was established (Fig. 1). It includes (i) identification of naturally present micro-organisms and residual bioburden, (ii) controlled contamination of vaginal tissue blocks with confirmation test, (iii) histological assessment of ECM architectural damage; (iv) quantitative efficacy validation of sterilization.

This experimental design ensures initial investigation of negative controls of donated vaginal wall tissue for naturally occurring micro-organisms and potential bacterial and fungal infection by blood agar stamps, and by plating of homogenized vaginal tissue on blood agar. Next, a controlled contamination was performed with micro-organisms commonly found in the human vagina microbiota. Controlled contamination was performed with one micro-organism per sample and confirmed by plating of the last wash medium on blood agar, and plating of homogenized tissue on blood agar. Tissue samples were then exposed to our sterilization methods. As extensive ECM damage can be inflicted during sterilization, the structural architecture of samples was assessed by Haematoxylin and Eosin (H&E) staining. Finally, sterilized test samples were stamped on blood agar and homogenized tissue was plated on blood agar to test. Residual micro-organisms from controlled contamination were quantified (number of colony-forming units per material) and identified by mass spectroscopy. All experiments were conducted thrice.

### Sterilization

Three sterilization methods were developed. These are based on (i) our original decellularization protocol^2^, (ii) our decellularization protocol combined with PAA/H_2_O_2_ treatment and (iii) our decellularization protocol with addition of a 1% and 10% mixture containing penicillin, streptomycin and amphotericin-B, and a final washing step with 70% ethanol.

#### Controlled contamination

A controlled contamination of the human vaginal wall tissue was performed. The selected bacterial (aerobic or anaerobic, gram^+^ or gram^−^) and fungal strains are commonly found in the healthy and diseased vaginal microbiota. Full thickness tissue blocks of 5 by 5 mm^2^ were inoculated prior to sterilization and decellularization by submersion in 1 mL solution of approximately 10^8^–10^9^ CFU. This was performed to achieve a minimum attachment of *L. crispatus* (anaerobic Gram+), *S. aureus* (aerobic Gram+, ATCC 49230), *E. coli* (aerobic Gram−, ATCC 25922) or *C. albicans* at 10^6^ CFU per strain, to allow assessment of the sterilization efficiency based on the 6 log-reduction criterium (see “Sterilization by antibiotics and antimycotics treatment” section) and to test worst-case contamination scenarios. The differences in controlled contaminant concentrations are due to limitations on the quantity of cultured CFUs that could be acquired at experiment initiation, and differences in optical density and the surface attachment ability. The added contaminant was allowed to attach to human vaginal wall blocks for 3 h at 37 °C in Dulbecco’s Modified Eagle Medium (DMEM—Gibco, ThermoFisher Scientific) with constant agitation at 120 rotations per minute (RPM). Three washes of 5 min at 37 °C and 120 RPM were performed in sterile PBS to remove non-adhered, added contaminants. Control groups without controlled contamination were included to assess resident microbiota of the samples and to serve as a reference to assess the efficacy of the respective sterilization method to eliminate the microbiota. All individual samples of all groups were homogenized in 500 µL of 0.5% Tween 80 (Sigma-Aldrich) in PBS using 2 mm zirconia beads (Biospecs) for 2 times 1 min at 7.000 RPM in a MagNA Lyser (Roche). Dilutions of the homogenate were cultured on blood agar (Colombia agar containing 5% sheep blood, ThermoFisher Scientific) and, depending on the microbial species, incubated for 1–7 days at 37 °C under appropriate aerobic or anaerobic conditions. All blood agar plates were quantitatively assessed by visual examination for the number of colonies that were formed. Controlled contamination was performed with tissue from five donors and measured in triplicates (total *n* = 15 samples per sterilization method).

#### Sterilization by decellularization [DC]

After controlled contamination, decellularization was initiated by 24 h incubation at 37 °C with constant agitation (100 rotations/min) in 0.18% w/w Triton x-100 (Sigma-Aldrich) and 0.015% w/w sodium-deoxycholate (Sigma-Aldrich) in PBS^93^. Tissue was washed twice for 20 min in PBS, followed by a 72 h wash at 4 °C with constant agitation in PBS with 1% PS (100 U/mL penicillin and 100 µg/mL streptomycin). Enzymatic digestion involved 24 h incubation at 37 °C with DNase I (150 IU/mL; Sigma-Aldrich) and 50 mmol MgCl_2_ (Sigma-Aldrich) in PBS with constant agitation (100 rotations/min). Tissue was washed twice for 20 min in PBS. A 24 h incubation at 4 °C was performed with constant agitation in PBS with 1% PS, followed by twice 20 min in PBS at room temperature with constant agitation (100 rotations/min). The duration of the total protocol was 6 days. All solutions were sterilized before use with a 0.22 μm sterile membrane filter (Merck Millipore). A non-sterilized control group was included.

#### Sterilization by peracetic acid/hydrogen peroxide treatment [PAA/H2O2-sterilization]

Decellularization was performed as described above in “Sterilization by decellularization [DC]” section. After enzymatic digestion, tissue was washed twice in PBS for 20 min each. Tissue was treated with PAA, acetic acid (AcA) and H_2_O_2_ in a 1:1:1 ratio. Several sterilization conditions were tested, which included concentrations of 0.01, 0.05 or 0.1% PAA/H_2_O_2_, sterilization durations of 2–3 h, and constant agitation at either 125 or 250 rotations/min. These solutions were prepared by diluting a PAA-mixture (Neodisher endo SEPT PAC, Dr. Weigert) 25 times in 100% ethanol, with further dilution in PBS to respectively 0.96%, 4.8% or 9.6% ethanol. A PAA/H_2_O_2_ concentration range was tested to minimize structural damage with sufficient sterilization action, and a control group with no PAA/H_2_O_2_ sterilization was included. Tissue was washed twice for 20 min in PBS. A 24 h incubation at 4 °C was performed with constant agitation in PBS with 1% PS, followed by twice 20 min in PBS at room temperature with constant agitation (100 rotations/min). The duration of the total protocol was 6 days. All solutions were sterilized before use with a 0.22 μm sterile membrane filter (Merck Millipore).

#### Sterilization by antibiotics and antimycotics treatment [AAE-based]

After controlled contamination, a 1 h wash was performed on a rollerbench at room temperature in PBS containing high concentrations of antimicrobials, i.e., 10% PS (1000 U/mL penicillin and 1,000 µg/mL streptomycin; Life Technologies Europe BV) and 10% amphotericin-B (25 µg/mL, Bio-Reagent, Sigma-Aldrich). Next, decellularization was performed in accordance to “Sterilization by decellularization” Sect^94^. However, for this protocol 1% PS washing solutions were supplemented with 1% amphotericin-B. After AA incubation, tissue was washed twice for 20 min in PBS, followed by 5 min in 70% ethanol and again twice in PBS for 20 min at room temperature with constant agitation (100 rotations/min). During experiments, DVM was soaked in 70% ethanol for 5 min to ensure adequate effect. Lastly, a 24 h incubation at 4 °C was performed with constant agitation in PBS with 1% PS and 1% amphotericin-B, followed by twice 20 min in PBS at room temperature with constant agitation (100 rotations/min). A control groups without decellularization was included. The duration of the total protocol was 7 days. All solutions were sterilized before use with a 0.22 μm sterile membrane filter (Merck Millipore).

#### Embedding by semi-automated tissue processing

Tissues were dehydrated and embedded in paraffin using the Epredia™ Excelsior™ AS Tissue Processor (Thermo Fisher Scientific). This sequence involved 1 h in 70% ethanol, 1 h in 90% ethanol, 1 h in 96% ethanol and thrice for 1 h in 100% ethanol at RT. Three sequential xylene steps at 37 °C, 40 °C and 45 °C were performed, followed by sequential paraffin baths 1, 2 and 3 in three steps of each 1 h and 20 min at 62 °C. Next, tissue was manually embedded in liquid paraffin of 55 °C by 1 h solidification at RT on a cooled plate of − 5 °C. After overnight 4 °C paraffin wax hardening, samples are ready for microtome sectioning.

#### Haematoxylin with eosin staining

Samples were fixed overnight at RT with constant agitation (on a roller) in 4% w/v formaldehyde (ROTI^®^ Histofix 4%; Carl Roth). Tissue was washed 30 min in PBS, followed by three cycles of 30 min in 70% ethanol (Sigma-Aldrich) at RT. Tissue was embedded according to “Embedding by semi-automated tissue processing” (above). Samples were sliced to 5 μm-thick sections using a microtome with a 6° knife angle. Sections were mounted on microscope slides in a water bath at 37.2 °C. The slide-sections were deparaffinized twice for 5 min in xylene, followed by hydration for 2 min in 100% ethanol (twice), 96% ethanol and 70% ethanol. Slides were H&E stained at RT in 1 mg/mL of Haematoxylin (Sigma-Aldrich) in 70% ethanol and 5 mg/mL of Eosin (Sigma-Aldrich) in 70% ethanol. Slides were embedded with 1x Entellan (Sigma-Aldrich) and dried overnight before inspection with the Leica DM5000B (Leica Biosystems) fluorescence microscope. H&E staining was performed on triplicates from three donors (*n* = 9 samples per sterilization method).

#### Assessment of sterilization efficacy

Sterility was evaluated conform the bioburden 6-log reduction criteria and in accordance with the ISO sterility guidelines, WHO-guidelines, guidelines of the European Pharmacopoeia 2.6.1 and the international code of practice^95,96^. In line with these guidelines, sterilization efficacy was quantified by plating of tissue homogenates on blood agar plates. After controlled contamination and sterilization, test samples were stamped on blood agar and then homogenized in 500 µL of PBS containing 0.5% Tween 80 (Sigma-Aldrich) using 2 mm zirconia beads (Biospecs) twice for 1 min at 7.000 RPM in a MagNA Lyser (Roche). The homogenates, and 10-fold serial dilutions thereof in PBS, were cultured on blood agar and incubated at 37 °C under aerobic and anaerobic conditions. Incubation was performed for 7 days for controls without controlled contamination, for 24 h after controlled contamination with *S. aureus* and *E. coli*, and for 48 h after controlled contamination with *L. crispatus* and *C. albicans*. The tissue residue after homogenization was placed in liquid TSB medium, and tubes were incubated at 37 °C shaking at 120 RPM for identical periods as the corresponding blood plates. All blood agar plates were quantitatively assessed by visual examination for the number of colonies that were formed. For assessment of the efficacy, successful sterilization was defined as complete extermination of all controlled contaminants with at least a 6-log reduction. TSB cultures were visually inspected and an aliquot of 10 µl was plated on blood agar plates in case of visible growth. Assessment of sterilization efficacy was performed on triplicates from three donors (*n* = 9 samples per sterilization method).

#### Identification of endogenous micro-organisms in tissue samples

Identification of endogenous micro-organisms in unprocessed vaginal wall tissue and in vagina wall tissue after controlled contamination and sterilization was carried out by plating of the last washing solutions on blood agar using a streak plate procedure and incubation for 24–48 h at 37 °C. After incubation, micro-organism identification was performed by mass spectrometry by a Bruker Biotyper MALDI-TOF MS system (Bruker Daltonics). Identification of endogenous micro-organisms was conducted on triplicates from three donors (*n* = 9 samples per sterilization method).

### Statistical analysis

The GraphPad Prism 10.2.3 (GraphPad Software) and Microsoft Office Excel 2019 (Microsoft Corporation) software for Windows were used for statistical analysis of the results, to identify any significant differences between inoculated controls and test specimens after sterilization. Homogeneity of variances was analyzed using the Barlett’s test. Sterilization results were then analyzed using the two-sample t-test with pooled variance, or by the Welch’s t-test in case of unequal variances. Data was plotted as mean ± standard deviation (SD). An obtained statistically significance was defined in terms of the following P-value: *for *P* ≤ 0.05, **for *P* ≤ 0.01 and ***for *P* ≤ 0.001.

## Results

### Controlled contamination and naturally occurring micro-organisms

**Fig. 2:**
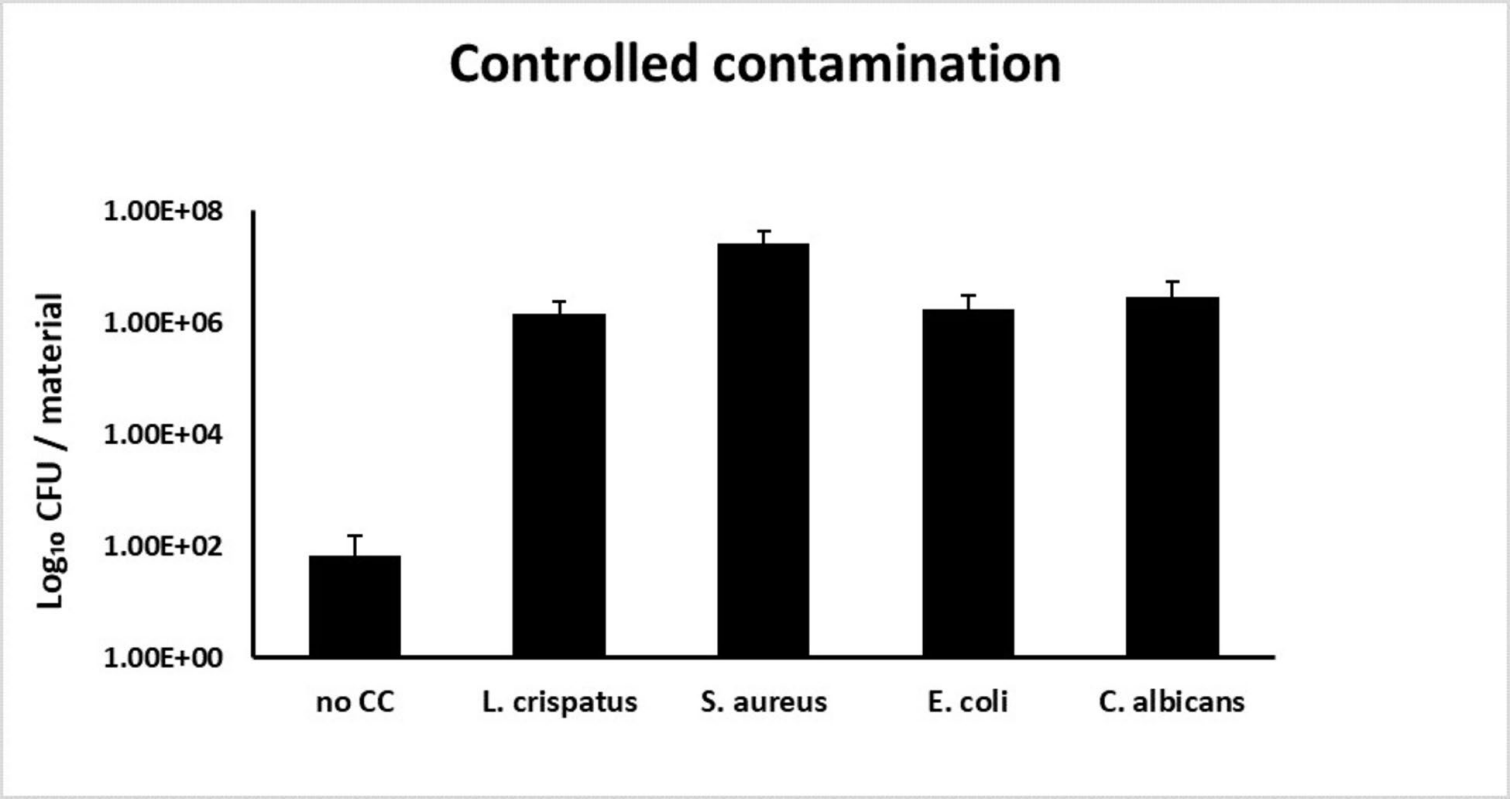
By quantification, controlled contamination with *L. crispatus*, *S. aureus*, *E. coli* and *C. albicans* strains was confirmed. After 3 h of incubation with inoculum, the number of colony-forming unit (CFU) per material was assessed. Depicted mean values (*n* = 15, triplicates from 5 donors) are 5.3 × 10^1^, 1.4 × 10^6^, 2.6 × 10^7^, 1.8 × 10^6^ and 2.9 × 10^6^ CFU/material, respectively.

Quantitative assessment of applied contaminants (Fig. 2) confirmed a mean adhesion of 1.4 × 10^6^ CFU/material, 2.6 × 10^7^ CFU/material, 1.8 × 10^6^ CFU/material and 2.9 × 10^6^ CFU/material of added contaminants per human vaginal wall tissue sample for either *L. crispatus*, *S. aureus*, *E. coli* or *C. albicans*, respectively. A negative control without controlled-contamination demonstrated the presence of 4 × 10^1^ CFU naturally occurring micro-organisms per human vaginal wall tissue sample.

Conditions of the AAE-sterilization protocol were pre-determined by controlled contamination experiments. For 1 h incubation with PS and amphotericin-B (Supplement 1), effective elimination of controlled bacterial contamination and largely effective elimination of added fungal contaminants was only seen for 10% AA concentrations. *C. albicans* was reduced from 1 × 10^7^ CFU/material to 17 CFU/material and from 1 × 10^8^ CFU/material to 40 CFU/material, thereby demonstrating a 6 log-reduction. Therefore, an AA concentration of 10% was considered sufficient. Concentrations of the digestion medium were tested for 24 h incubation (Supplement 2), and effective treatment was found at 100% of the DC concentrations. Furthermore, for 24 h incubation with PS and amphotericin-B (Supplement 3), effective removal of controlled bacterial and fungal contamination was seen at concentrations of 1%. Lastly, effective removal of controlled bacterial and fungal contaminants with 70% ethanol (Supplement 4), was seen within 2 min of incubation.

### Micro-organism identification in native and processed vaginal tissue

In donated tissue from healthy masculine transgender individuals, anaerobic gram^+^ pathogens and micro-organisms were most common. A high variability was observed (Table 1; Supplement 5), but most micro-organisms found in native controls are part of the normal, healthy microbiota of the human vagina. These were *Gardnerella vaginalis*, *Prevotella timonensis*, *Finegoldia magna*, *Winkia neuii*, *Peptoniphilus lacydonensis*, *Peptoniphilus lacrimalis*, *Streptococcus agalactiae* and *Staphylococcus lugdunensis*^39,40,42,44,97^. Furthermore, the rare species *Facklamia hominis* was identified, with the vagina (reported as female genitourinary tract) suggested as its natural habitat^98^. *Anaerococcus vaginalis* and *Corynebacterium tuberculostericum* were further found and are generally less prominent in the vaginal microbiota, but have been reported as part of the vaginal microbiota in masculine transgender individuals due to long-term androgen exposure^99^. Pathogens detected in native controls were *Cutibacterium acnes*, *Cutibacterium avidum*, *Propionimicrobium lymphophilum*, *Actinomyces timonensis* and *Bacillus cereus / thuringiensis*. Upon decellularization, only controlled contaminants were observed but no native microbes. Upon AAE-based sterilization, controlled contaminants as well as native bacteria (both pathogens and commensals) were eliminated.

**Table 1:** Identification of resident micro-organisms in native vagina wall tissue, after decellularization and after decellularization with complementation of an AAE-based sterilization treatment.

Sterilization method	Resident micro-organisms	Aerobic/anaerobic	Gram	Vagina microbe
Native control	*Winkia neuii*	Aerobic	+	Yes
	*Corynebacterium tuberculostearicum*	Aerobic	+	Yes, in masculine transgenders
	*Prevotella timonensis*	Anaerobic	−	Yes
	*Gardnerella vaginalis*	Anaerobic	+	Yes
	*Finegoldia magna*	Anaerobic	+	Yes
	*Streptococcus agalactiae*	Anaerobic	+	Yes
	*Staphylococcus lugdunensis*	Anaerobic	+	Yes
	*Peptoniphilus lacydonensis*	Anaerobic	+	Yes
	*Peptoniphilus lacrimalis*	Anaerobic	+	Yes
	*Anaerococcus vaginalis*	Anaerobic	+	Yes, in masculine transgenders
	*Facklamia hominis*	Anaerobic	+	Suggested
	*Propionimicrobium lymphophilum*	Anaerobic	+	No
	*Cutibacterium acnes*	Anaerobic	+	No
	*Cutibacterium avidum*	Anaerobic	+	No
	*Bacillus cereus/thuringiensis*	Anaerobic	+	No
	*Actinomyces timonensis*	Anaerobic	+	No
Decellularization	None	NA	NA	NA
Sterilization AAE + 70% ethanol	None	NA	NA	NA

Only bacteria were identified and therefore distinguished based on aerobic/anaerobic nature, Gram positive/negative character and their vagina microbiota property. No resident pathogens were detected after decellularization, or after decellularization with complementary AAE-based sterilization.

*NA* not applicable.

### Assessment ECM structure of sterilized DVM

**Fig. 3:**
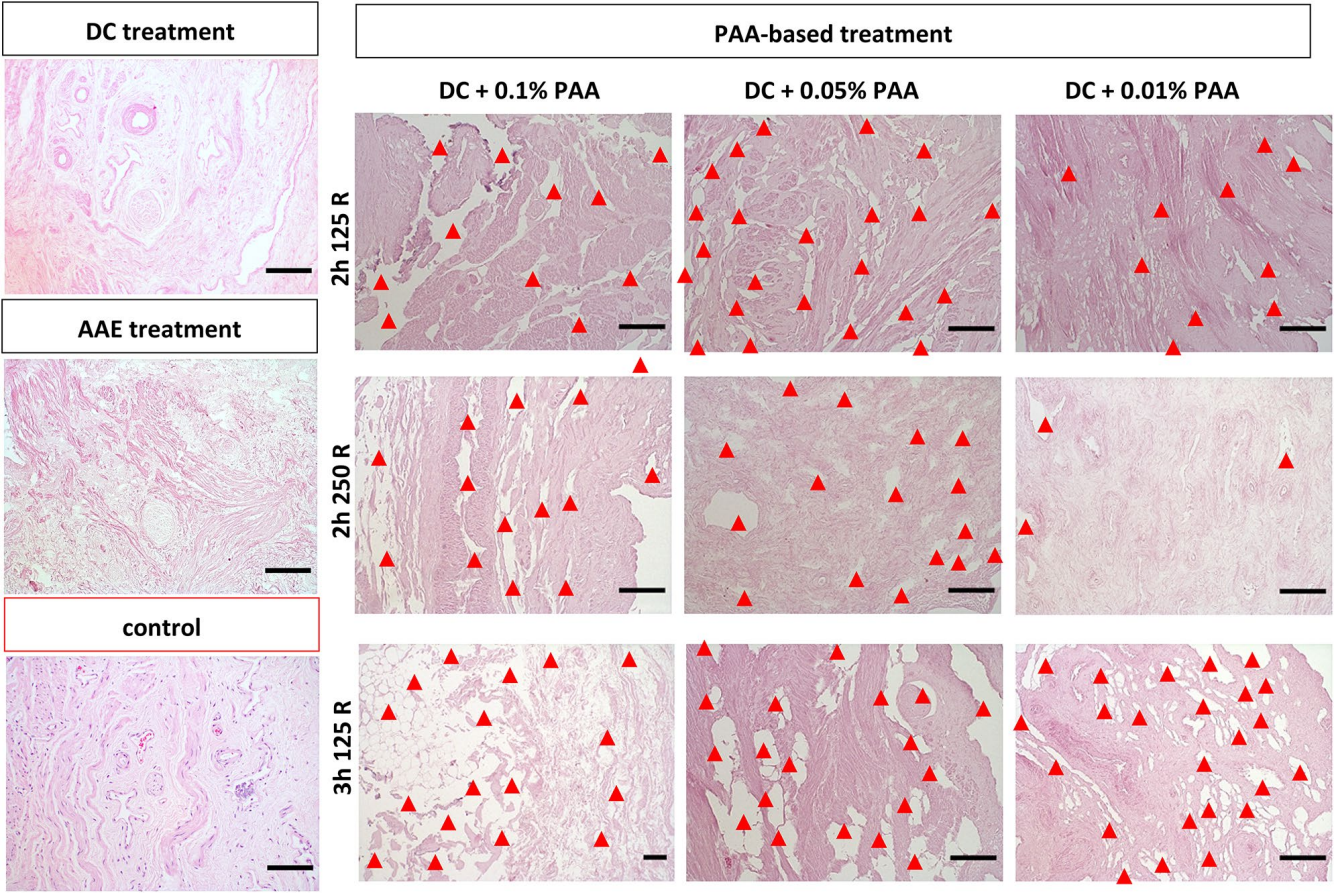
Structural analysis of decellularized vaginal wall matrix after chemical decellularization (DC) only, sterilization with 0.1%, 0.05% or 0.01% PAA/H_2_O_2_ treatment and AAE-based sterilization. Histological staining with Haematoxylin and Eosin (H&E) demonstrates damage to the ECM structure (red arrowheads) for sterilization with 0.1%, 0.05% or 0.01% PAA/H_2_O_2_ for 2 h sterilization at 125 rotations/min or 250 rotations/min constant agitation and for 3 h sterilization at 125 rotations/min, compared to a decellularized vaginal wall control sample without exposure to a sterilization procedure. Scalebars are 100–200 μm in length. Depicted histological images are a representation of triplicates from 3 donors (*n* = 9 per test condition). Scalebars are 100 μm in size.

The ECM architecture was investigated by histological assessment after sterilization (Fig. 3). After decellularization, the ECM architecture was well preserved. The ECM architecture was further assessed for potential damaging effects by PAA/H_2_O_2_-sterilization for various concentrations of 0.01%, 0.05% and 0.1%. Figure 3 demonstrates that the ECM structure of decellularized vaginal wall tissue is diminished for sterilization with all of these PAA/H_2_O_2_ concentrations. Furthermore, with respect to PAA/H_2_O_2_-sterilization of various durations and agitation frequencies, the ECM architectural damage is visible after both 2 h and 3 h of sterilization at either 125 or 250 rot/min, as clear holes or completely dissolved tissue. Damage is more prominent for increased PAA/H_2_O_2_ concentrations and longer sterilization times. Histological assessment further demonstrated a maintained ECM architecture after AAE-based treatment.

### Assessment sterility treatment by decellularization

**Fig. 4:**
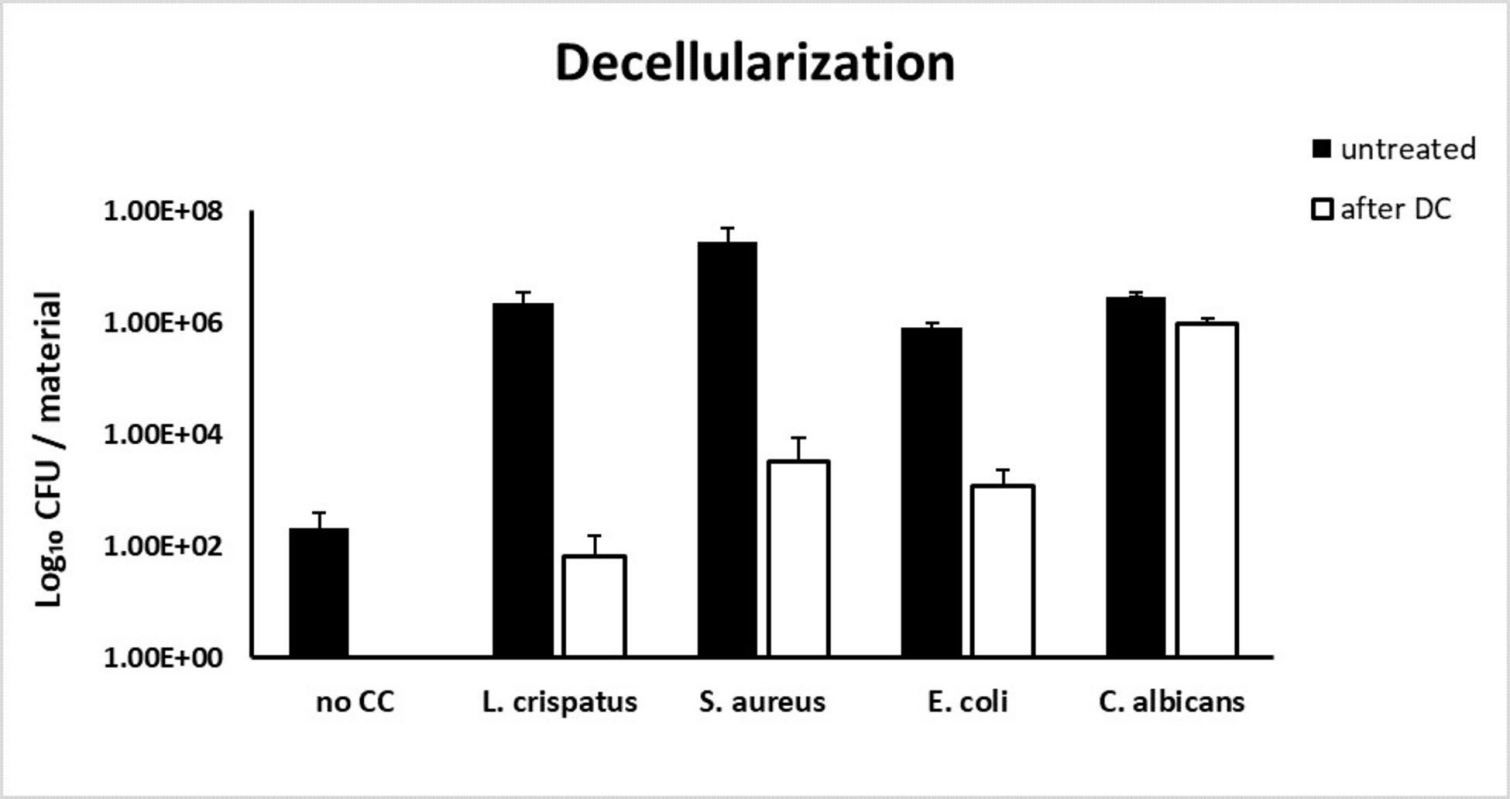
Controlled contamination with *L. crispatus*, *S. aureus*, *E. coli* and *C. albicans* strains was performed and the number of colony-forming unit (CFU) per material was assessed for untreated specimen and after chemical decellularization (*n* = 3, triplicate from 1 donor). No significant elimination of contaminants was observed.

**Fig. 5:**
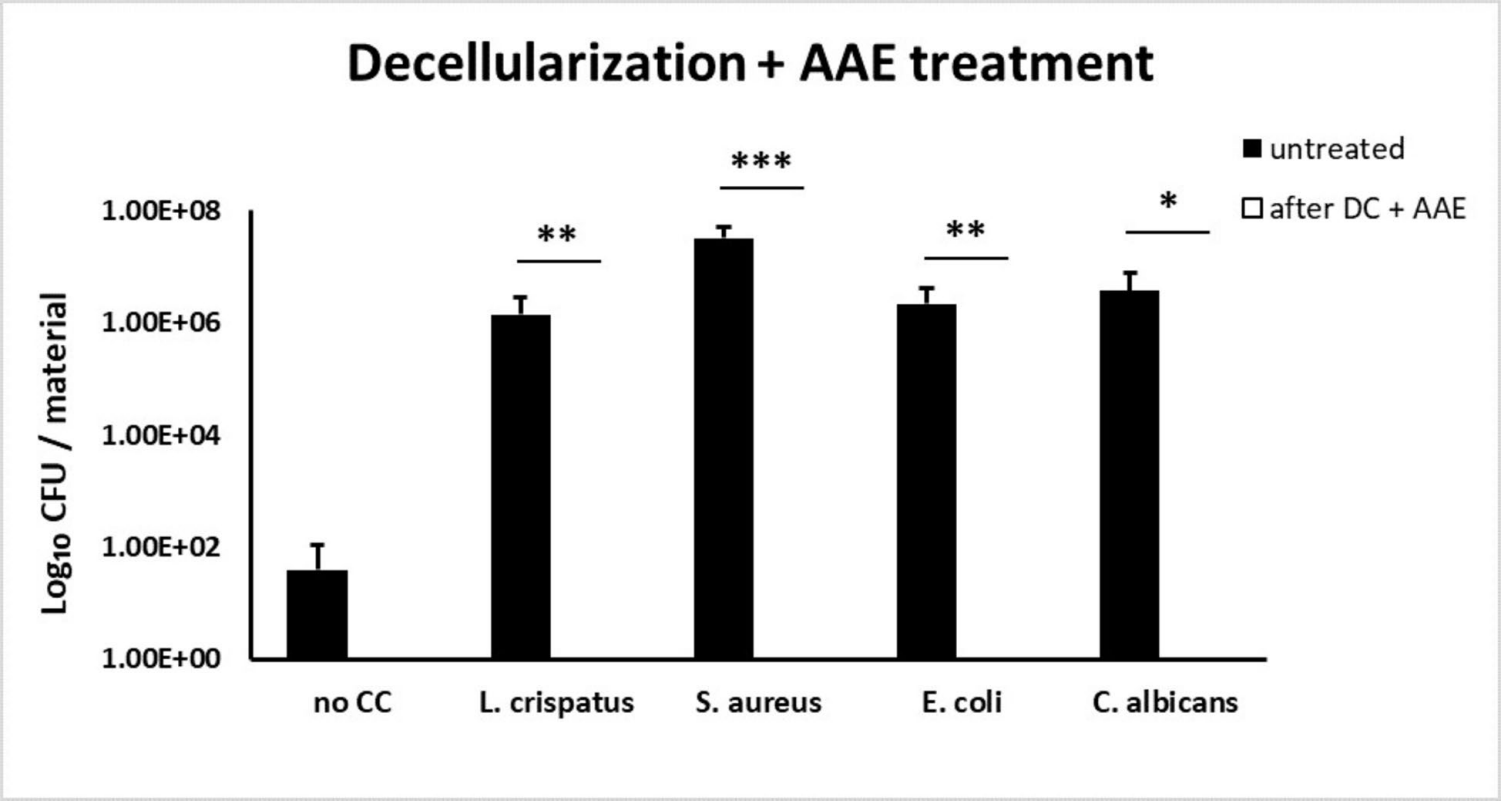
Controlled contamination with *L. crispatus*, *S. aureus*, *E. coli* and *C. albicans* strains was performed and the number of colony-forming unit (CFU) per material was assessed for untreated specimen and after chemical decellularization with complementary AAE-based sterilization treatment (*n* = 9, triplicates from 3 donors). A significant elimination of contaminants was observed for species of *L. crispatus* (*P* < 0.01), *S. aureus* (*P* < 0.001), *E. coli* (*P* < 0.01) and *C. albicans* (*P* < 0.05).

The efficiency of our sterilization methods was quantified after decellularization (Fig. 4) and for the AAE-based treatment (Fig. 5). Efficiency of PAA/H_2_O_2_-sterilization was not assessed because the major damage of the ECM structure would not allow translation to clinical settings. Chemical decellularization demonstrated no significant reduction of *L. crispatus*, *S. aureus*, *E. coli* or *C. albicans*. Although the quantitative reduction of *L. crispatus* and *S. aureus* did comply with the log-6 reduction criterium, this was insufficient for complete sterilization.

The sterilization effect of decellularization in combination with AAE-based treatment demonstrated complete eradication as judged from lack of growth on both the agar plates and the liquid TSB cultures of *L. crispatus* (*P* < 0.01), *S. aureus* (*P* < 0.001), *E. coli* (*P* < 0.01) and *C. albicans* (*P* < 0.05), which is in compliance with the log-6 reduction criterium.

## Discussion

### Sterilization of vagina wall tissue and the need for efficacy validation

Approximately 90,000 transplantations are performed worldwide each year, yet a donor organ shortage remains^20^. Recently, tissue engineering and regenerative medicine have resulted in promising new decellularized scaffolds for tissue replacement of various organs, including the vagina. Moreover, the chance of graft survival has improved by preventing tissue rejection through immunosuppression therapy. However, clinical application of tissue engineered transplants in immunocompromised hosts is challenging, as their diminished inflammatory response makes them more susceptible to infections. Therefore, risk of transplant transmitted diseases is of considerable concern. Guidelines and methods for microbiological screening of transplants vary across countries and even national regions^100^. Despite the official registration of transplant-transmitted infections, only a 1% occurrence is reported as this is generally only recognized after clusters of infections are observed in recipients with a common donor. However, in case of transmitted infections, the risks are severe and include sepsis, graft rejection, and postoperative morbidity and mortality^20^. Therefore, success of vagina transplantation relies on the formulation and utilization of an effective sterilization procedure prior to implantation.

To reliably confirm efficient sterilization, validation is required by controlled contamination at high bioburden concentrations to simulate worse-case scenarios. High bioburdens on the scaffold can either originate from high endogenous microbe concentrations in the donor tissue, or from later contamination by micro-organisms that are resistant to specific disinfection or sterilization approaches. Quality control for sterility validation should include both identification (qualification) and quantification of potential contaminants, to allow risk assessment and any further optimization to guarantee successful sterilization before implantation.

In general, testing of mass-produced biomaterials for sterility validation is performed through large quantity, random sampling with complete sample loss. Sterility assessment of large-size biomaterials typically involves the constant removal, and consequent loss, of a biomaterial section from one sample. For biomaterials that are manufactured at small quantities and volumes, like our matrix, both approaches would not be feasible. Under these conditions, the European Pharmacopeia guidelines allow sterility testing on the final washing or storage solution. In this study, this last form of sterility testing was conducted.

### Aim and study design in accordance to official guidelines

This study aimed to compare three of our tailored sterilization methods for safe implantation of decellularized human vagina wall tissue in future clinical applications. Sterilization efficacy was evaluated in accordance to internationally established guidelines^95,96^, the bioburden reduction criteria (6-log numbers of CFU reduction and ISO 14973) for residual microbes and contaminants at low tissue quantity and volume, and preservation of the ECM structure. Although over the years, various decellularization techniques for vaginal tissue have been designed, tissue disinfection is often not considered, is outside the scope of the particular study, or lacks validation. Standard chemical and physical sterilization approaches consist of antibiotics, antimycotics, PAA, ethanol, freeze-drying and/or gamma-irradiation^29,35–37^. For vagina reconstruction from dermis, polyglycolic acid-poly(lactic-co-glycolic acid), poly(L-lactide-co-caprolactone) and amniotic membrane, this has involved freeze-drying, gamma-irradiation and EO gas^101–104^. However, most studies did not assess sterilization efficacy nor the induced structural damage. Therefore, we aimed to perform a well standardized and complete study to develop a safe and reliable method to sterilize human decellularized vaginal scaffolds for vaginal reconstruction, and thereby bridging the gap towards their clinical application.

### Decellularization carries insufficient disinfection potential

We studied the sterilization effect of decellularization, which previously demonstrated to form complete acellular tissue with preservation of ECM architecture, suitable biomechanical tissue properties, and retained ECM proteins and vascular architecture^2^. Upon controlled contamination, DC did not affect fungal contamination and was incapable of complete bacterial elimination. All pathogenic and non-pathogenic micro-organisms of the native microbiota were present at low CFU quantities and eliminated by DC, but no endogenous fungi were present to demonstrate the anti-fungal action of DC. Of the identified endogenous micro-organism, the *F. hominis* is rare, but considered a non-pathogen for the female genitourinary tract^98^. Both *A. vaginalis* and *C. tuberculostericum* are less prominent in the vagina, but are increased in the vaginal microbiota of masculine transgender individuals due to long-term androgen exposure. Furthermore, some bacterial infections were identified (*P. lymphophilum*, *C. acnes*, *C. avidum*, *(A) timonensis* and *(B) cereus/thuringiensis*). As DC demonstrated incapable of complete microbial contaminant removal and thereby does not comply with sterilization criteria, infections would be of concern especially for clinical application of this method, considering the associated risk for transplant transmitted diseases. Although other studies on tissue-engineered constructs have applied similar decellularization protocols for porcine aorta^93^, human muscle^105^, and rat vagina^29^, validation of any potential sterilization effects by a similar chemical DC protocol has to our knowledge not been previously reported. Regarding this limited data availability in the literature and prospective application of decellularized scaffolds, the insufficient sterilization effect of decellularization is a noteworthy finding.

### Additional PAA/H2O2-sterilization destructive to ECM structure

The second sterilization method consisted of the decellularization protocol a PAA/H_2_O_2_-sterilization. PAA is a well-known and broadly applied decontaminant, but has serious health and environmental risks. Among others, PAA contact is irreversible destructive to skin^106^ and eyes, its inhalation irritates nose^107^, throat and lungs, and animal studies further indicate neurotoxicity, reproductive toxicity and carcinogenic action^108^. Therefore, our medical center applies strict regulations regarding the use of PAA for experimental work. Consequently, we had to replace pure PAA by an endoscope disinfectant solution that consists of a mixture of PAA, acetic acid and H_2_O_2_ diluted 25 times at a 1:1:1 ratio in ethanol. PAA/H_2_O_2_-sterilization demonstrated considerable damage, manifested as clear holes or completely dissolved tissue in the histological sections, that increased with PAA/H_2_O_2_-concentration and incubation time. Tissue samples were thereby softened and became difficult to process. These findings are contrasting previous reports regarding 2 h PAA-treatment of acellular vaginal matrix with additional freeze-drying and gamma-irradiation^35^, and regarding non-destructive sterilization by 0.1% PAA of decellularized cardiac and arterial valves^60^, lung^61^, liver^62,63^, bladder^64–66^, spleen^67,68^, kidney^69,70^, small intestine^71–73^, tendon^74,75^ and nerve^76^. Furthermore, the absence of damage after similar decellularization and 0.1% PAA sterilization of porcine vagina^37^ and heart^109^, suggests that our observed ECM damage can be best explained by the combined exposure to PAA, H_2_O_2_, acetic acid and ethanol. Strong oxidation of PAA and the synergistic effect with acetic acid and hydrogen peroxide both destroy the cell membranes, as well as matrix proteins and enzymes^46^. Ethanol causes protein denaturation, destruction of enzymes and reduction of collagen content, and H_2_O_2_ oxidation affects the physical and chemical biomaterial properties^46^. We considered the observed ECM damage after our PAA/H_2_O_2_-sterilization unacceptable for future clinical applications even for short durations of 2 h and at low concentrations of 0.01%. Further biochemical quantification of ECM components (i.e., collagen, elastin, laminin, fibronectin, or glycosaminoglycans) could be used to determine the extent of damage and to validate our qualitative findings.

### Additional AAE-based treatment for effective sterilization

As third sterilization approach, the decellularization protocol was complemented with antibiotics, antimycotics and ethanol. This AAE-based sterilization combines action against aerobic and anaerobic species of Gram^+^ and Gram^−^ bacteria and fungi, and can further act against protozoa^38^. This last effect was not investigated in this study. Our AAE-based protocol demonstrated efficient sterilization of controlled-contaminated human vagina wall tissue without any visual destructive effects on the ECM structure. Similar decellularization and sterilization of human vaginal tissue^94^ without tissue damage has previously been reported, but without further validation of sterilization efficiency. Regarding our findings, this treatment carries potential for translation towards tissue banks and clinical settings, and for usage of our sterile DVM in future clinical applications.

### Limitations

Whether our AAE-based sterilization treatment impacts the biomechanical properties of vagina wall tissue or its ECM constituent proteins, has been outside the scope of this study but are fundamental for in vivo tissue regeneration and durability of the implant. Modifications to morphology and ECM components cause alterations in cell adhesion and cell-biomaterial interactions, and we have previously demonstrated that these ECM proteins are crucial to maintain elasticity. Therefore, the impact of AAE-based treatment and the action of residual sterilization agents on cell adhesion, cytotoxicity, preservation of ECM constituent proteins and tissue biomechanical properties will be addressed in an upcoming study.

In this study, controlled contamination was performed with excessive amounts (10^8^–10^9^ CFU/ml) of either *L. crispatus*, *S. aureus*, *E. coli* or *C. albicans* to test worse-case scenarios. These micro-organisms are representatives of common Gram^+^ and Gram^−^ bacteria, anaerobic bacteria and a fungal species of the microbiota of the human vagina. The significance of micro-organism elimination by AAE-based treatment was tested and confirmed by a two-sample t-test with pooled variance, or by the Welch’s t-test for unequal variance. Alternatively, the Wilcoxon rank sum w test can be performed as nonparametric variant to decrease sensitivity to big deviations from normality distribution and exclude the impact of heterogeneity of variances. To this end, we performed a second statistical analysis with the Wilcoxon rank sum w test, that confirmed significant elimination of *L. crispatus*, *S. aureus*, *E. coli* and *C. albicans* by the AAE-based treatment.

Although all controlled-contaminants and naturally occurring micro-organisms were successfully eliminated by our AAE-based treatment, this might not be the case for all bacteria, fungi and especially protozoa forming the vaginal microbiota. Despite the broad disinfection and sterilization range of ethanol, penicillin, streptomycin, amphotericin-B and our decellularization agents combined, we do advise validation testing to ensure elimination of, for example, infrequent human vagina commensals or pathogens, protozoa or combinations of multiple species at extremely high concentrations. Although ethanol is reported capable of inactivating enveloped viruses that includes influenza, Ebola and coronaviruses^110^, it is less or not effective against other viruses such as human papillomavirus (HPV) or acquired immunodeficiency virus (AIDS)^110,111^. There are currently no medical or natural treatments to cure HPV or AIDS^112,113^, therefore extensive testing prior to transplantation of human donor vagina wall tissue is required. Next, although elimination of *L. crispatus*, *S. aureus*, *E. coli* and *C. albicans* was achieved for endogenous micro-organisms combined with one controlled-contaminant, the AAE-based treatment might be less efficient for contaminations that occur in unison as is the case in the authentic vaginal microbiota. However, in light of the full removal of all endogenous micro-organisms combined with a worse-case controlled-contamination, we believe that AAE-based treatment will in most cases be sufficient for sterilization of donated vagina wall tissue.

We further want to emphasize that AAE-based treatment was validated for vagina wall tissue blocks of 5 by 5 mm^2^. We previously validated our decellularization protocol to be effective for tissue blocks, rings, and full organs. Together with the validation guideline principles, successful translation of our AAE-method to sterilization of complete organs is expected. Lastly, we want to caution for the misuse and overuse of antibiotics-based sterilization, as this can result in drug-resistant pathogens. Globally, antimicrobial resistant bacteria cause millions of deaths^114^. Furthermore, these resistant pathogens harm modern medicine by making infections harder to treat and thereby increasing the risks of medical procedures and treatments. In terms of economic costs, the yearly losses due to antimicrobial resistance is estimated at trillions of US dollars^114^. In our protocol, addition of ethanol will help prevent resistance development.

### Future perspective

Future investigations on human decellularized vagina wall need to be performed to unravel whether cell attachment or proliferation is impaired by the AAE-treatment and induces changes to the surface topography. Furthermore, changes in biomechanical properties due to modification of surface chemistry and texture or biomaterial integrity of sterilized decellularized vagina wall should be examined. These studies will provide insight into possible side effects caused by the AAE-based sterilization protocol. Moreover, prior to clinical application, a comprehensive evaluation of the ECM integrity is advised, including biochemical quantification of ECM components (such as elastin, collagen, fibronectin, laminin and glycosaminoglycans). Additionally, immunofluorescent imaging may be included to facilitate the evaluation of ECM component organization and preservation.

## Conclusion

This research demonstrated that treatment of human vaginal wall tissue by chemical decellularization has disinfection potential with preserved ECM structure, but with insufficient sterilization efficacy. Additional sterilization with 0.01%, 0.05% and 0.1% PAA/H_2_O_2_ resulted in major ECM damage, that is considered unacceptable for clinical settings. Our decellularization protocol combined with AAE-based treatment demonstrated effective in producing sterile tissue with histological preservation of the ECM structure. This method is suitable for translation into clinical settings, and could be used for sterilization of other biomaterials such as other acellular biological scaffolds, or even biomaterials of polymeric or metallic origin. Furthermore, the sterile decellularized vaginal scaffold developed here, carries potential for first transplantation of human vagina after future optimization of biochemical and biomechanical characteristics.

## Electronic Supplementary Material

Below is the link to the electronic supplementary material.

Supplementary Material 1

## Data Availability

Data is available upon request from the corresponding author.
