# Supplementary material for: A sterilization method for human decellularized vaginal matrices
Source: Sci Rep. 2024 Dec 30;14:31728. doi: 10.1038/s41598-024-82409-4 (PMC11685901; doi:10.1038/s41598-024-82409-4)
Supplement: Supplementary file 1 — Supplementary Material 1 [file 41598_2024_82409_MOESM1_ESM.docx]

# **Supplement 1: Effect 1h incubation with AA**

**­­­
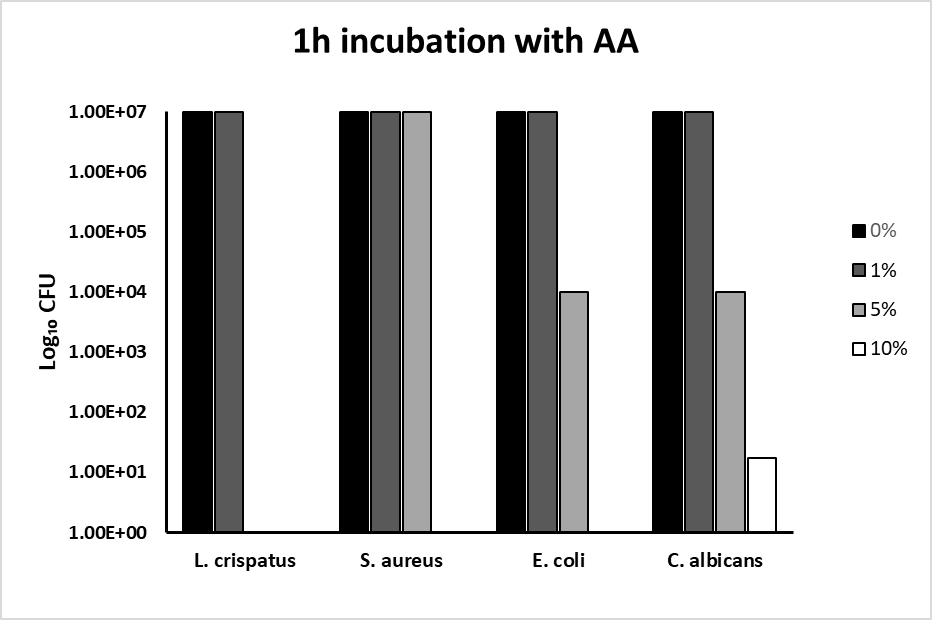
**

**
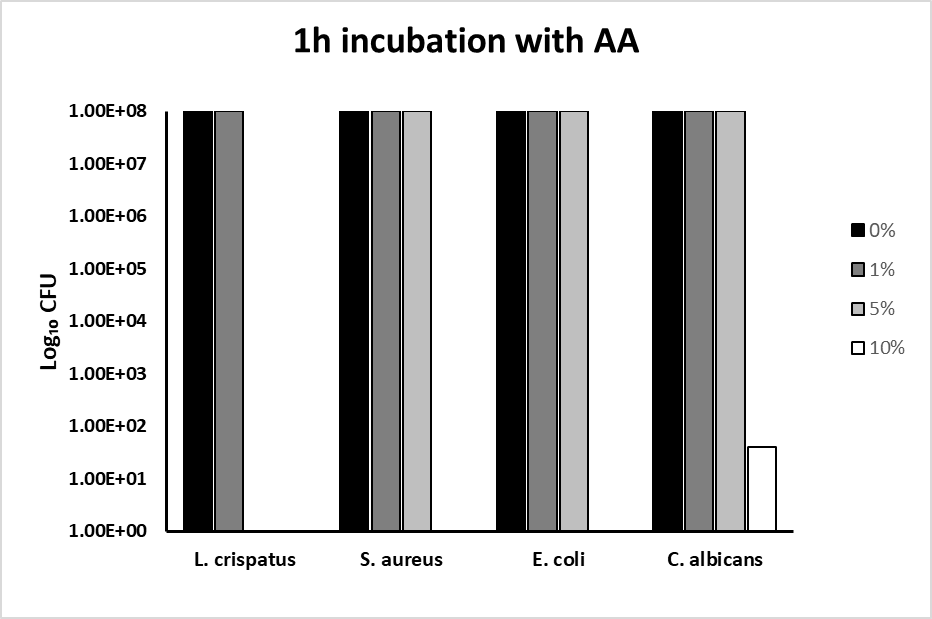
**

*Figure S1: Controlled contamination with L. crispatus, S. aureus, E. coli and C. albicans strains was performed and the number of colony-forming unit (CFU) per material was assessed for specimen treated by 1h incubation with 0, 1, 5 or 10% penicillin, streptomycin and amphotericin B [AA-solution] (n = 3, triplicates from 1 donor). A significant elimination of contaminants was observed for all species with a 10% AA-solution for controlled contamination with A) 10^7^ and B) 10^8^ CFU.*

# **Supplement 2: Effect 24h incubation with digestion medium**

**
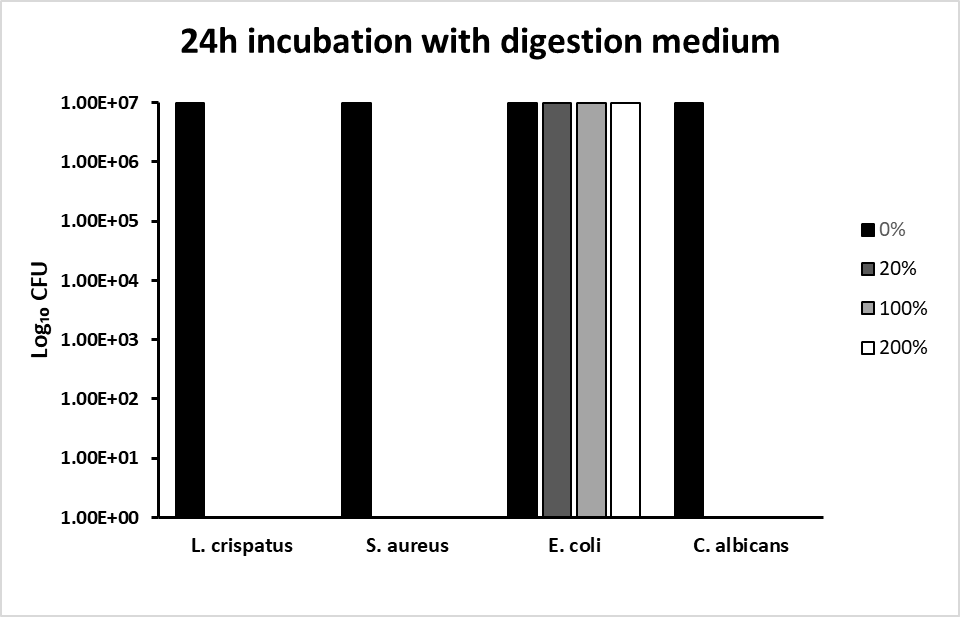
**

**
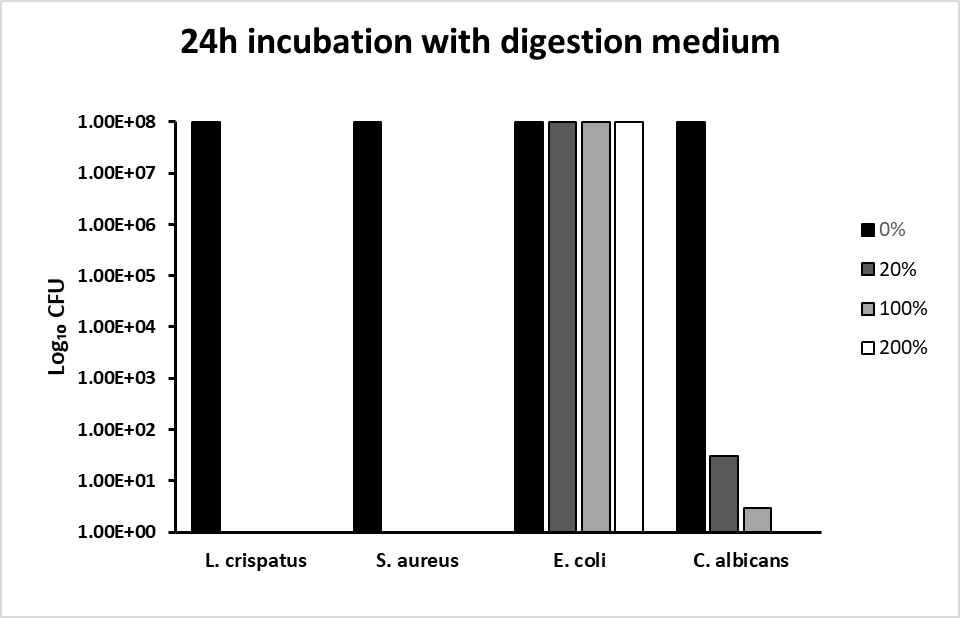
**

*Figure S2: Controlled contamination with L. crispatus, S. aureus, E. coli and C. albicans strains was performed and the number of colony-forming unit (CFU) per material was assessed for specimen treated by 24h incubation with 0, 20, 100 or 200% digestion medium (n = 3, triplicates from 1 donor). A significant elimination of contaminants was observed for all species except for E. coli with 100% and 200% digestion medium for controlled contamination with A) 10^7^ and B) 10^8^ CFU.*

# **Supplement 3: Effect 24h incubation with AA**


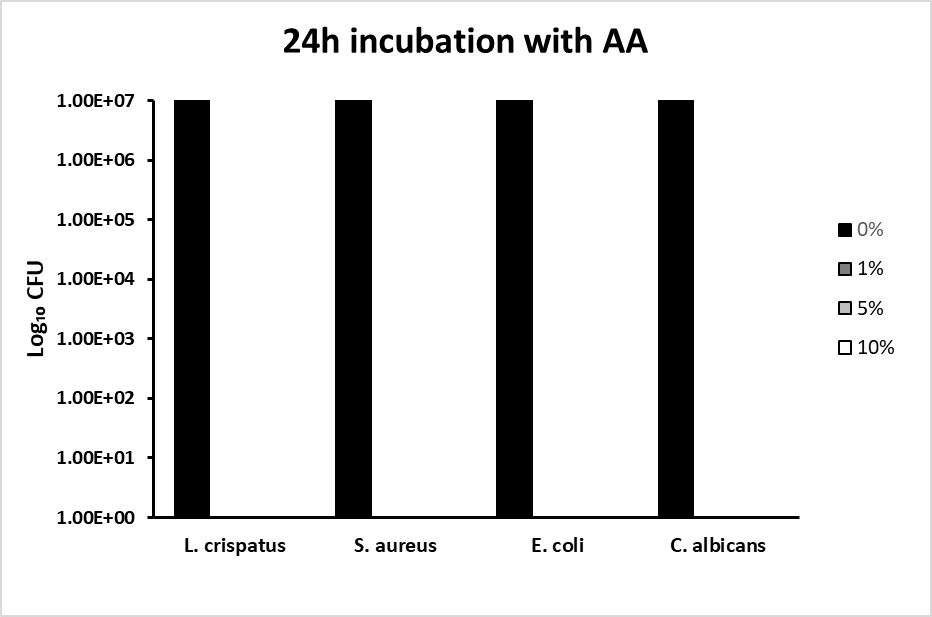


**
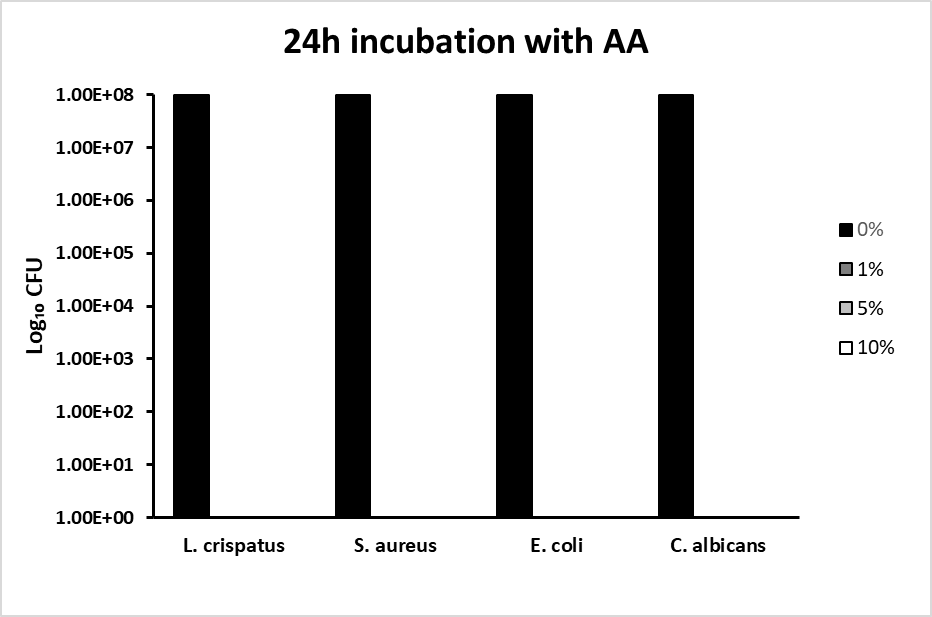
**

*Figure S3: Controlled contamination with L. crispatus, S. aureus, E. coli and C. albicans strains was performed and the number of colony-forming unit (CFU) per material was assessed for specimen treated by 24h incubation with 0, 1, 5 or 10% penicillin, streptomycin and amphotericin B [AA-solution] (n = 3, triplicates from 1 donor). A significant elimination of contaminants was observed for all species with a 1, 5 and 10% AA-solution for controlled contamination with A) 10^7^ and B) 10^8^ CFU.*

# **Supplement 4: Effect incubation with 70% ethanol**


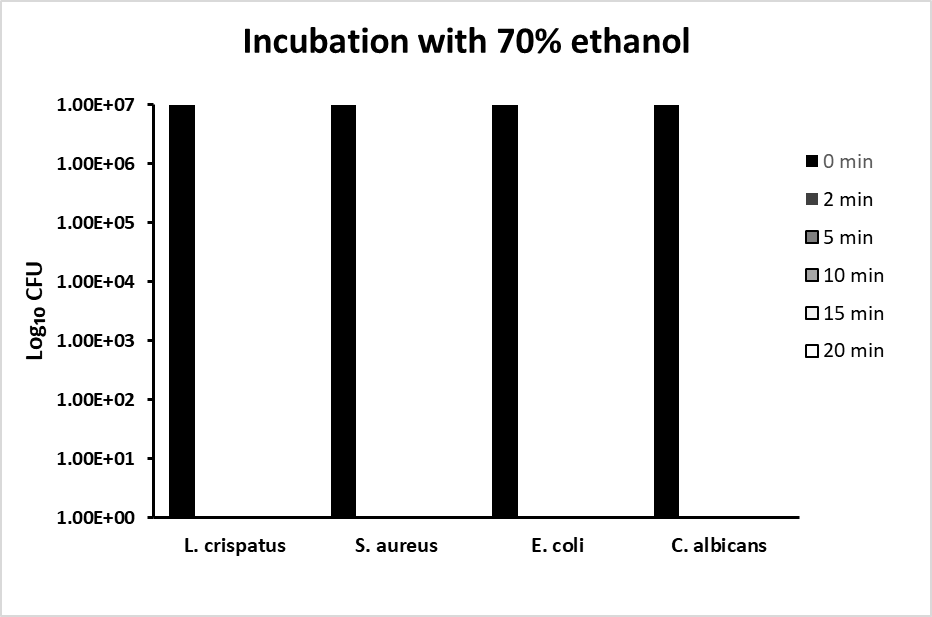

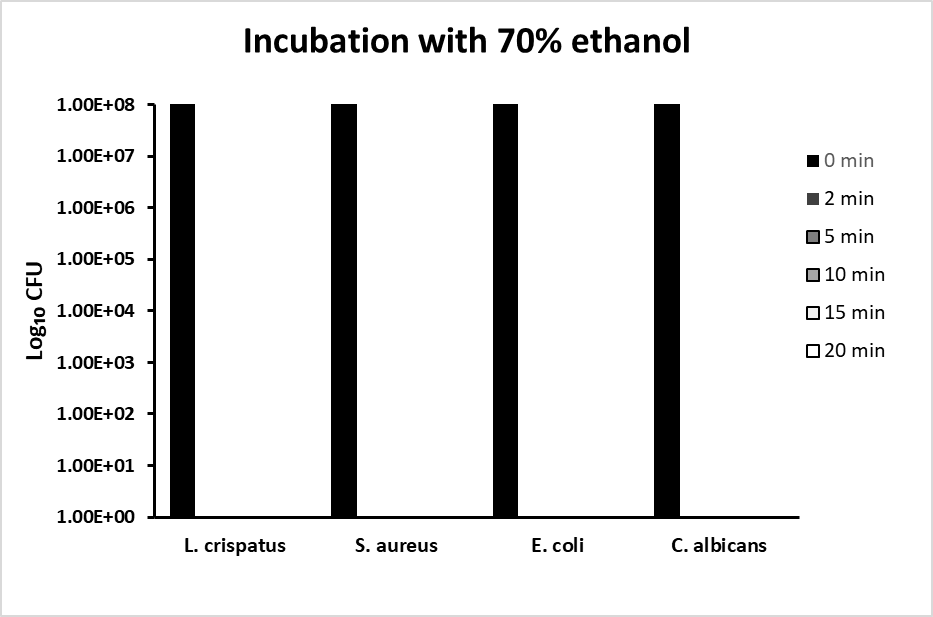


*Figure S4: Controlled contamination with L. crispatus, S. aureus, E. coli and C. albicans strains was performed and the number of colony-forming unit (CFU) per material was assessed for specimen treated by 0, 2, 5, 10, 15 or 20 minutes of incubation with 70% ethanol (n = 3, triplicates from 1 donor). A significant elimination of contaminants was observed for all species after 2, 5, 10, 15 and 20 minutes of incubation for controlled contamination with A) 10^7^ and B) 10^8^ CFU.*

# **Supplement 5: Frequency of identified endogenous micro-organisms**

*Table S5: quantification of identified endogenous micro-organisms in donor 1, 2 and 3. Supplemental information to Table 1.*

| Identified endogenous micro-organisms | Donor 1 | Donor 2 | Donor 3 |
| --- | --- | --- | --- |
| *Winka neuii* | x |  |  |
| *Corynebacterium tuberculostericum* | x |  |  |
| *Finegoldia magna* | x | x | x |
| *Streptococcus agalactiae* | x |  |  |
| *Gardnerella vaginalis* | x |  |  |
| *Cutibacterium acnes* | x | x | x |
| *Staphylococcus lugdunensis* | x |  |  |
| *Cutibacterium avidum* | x |  |  |
| *Actinomyces timonensis* |  | x |  |
| *Facklamia hominis* |  | x |  |
| *Prevotella timonensis* |  | x | x |
| *Peptoniphilus lacrimalis* |  | x |  |
| *Propionimicrobium lymphophilum* |  |  | x |
| *Anaerococcus vaginalis* |  |  | x |
| *Peptoniphilus lacydonensis* |  |  | x |
| *Bacillus cereus / thuringiensis* |  |  | x |
